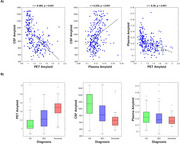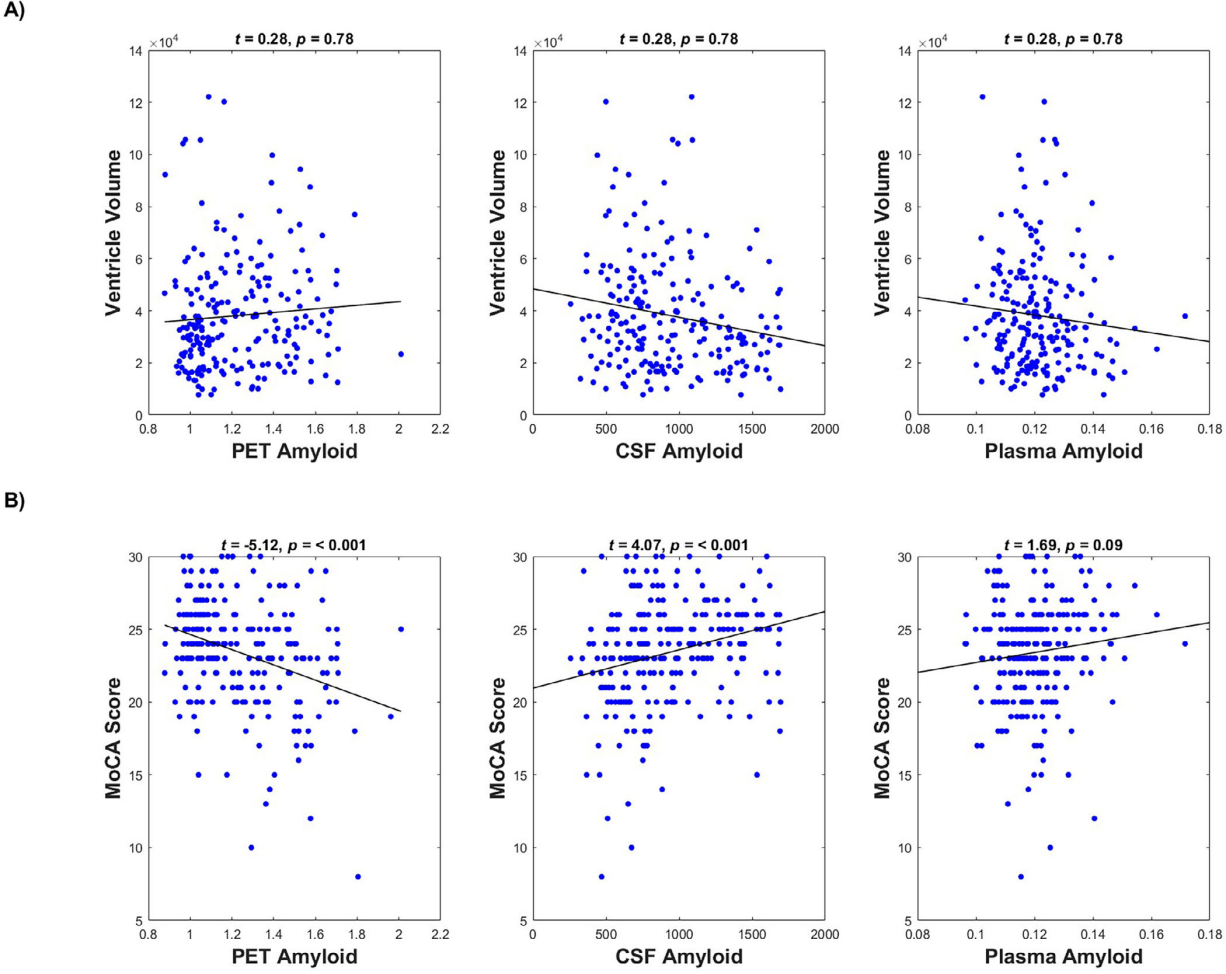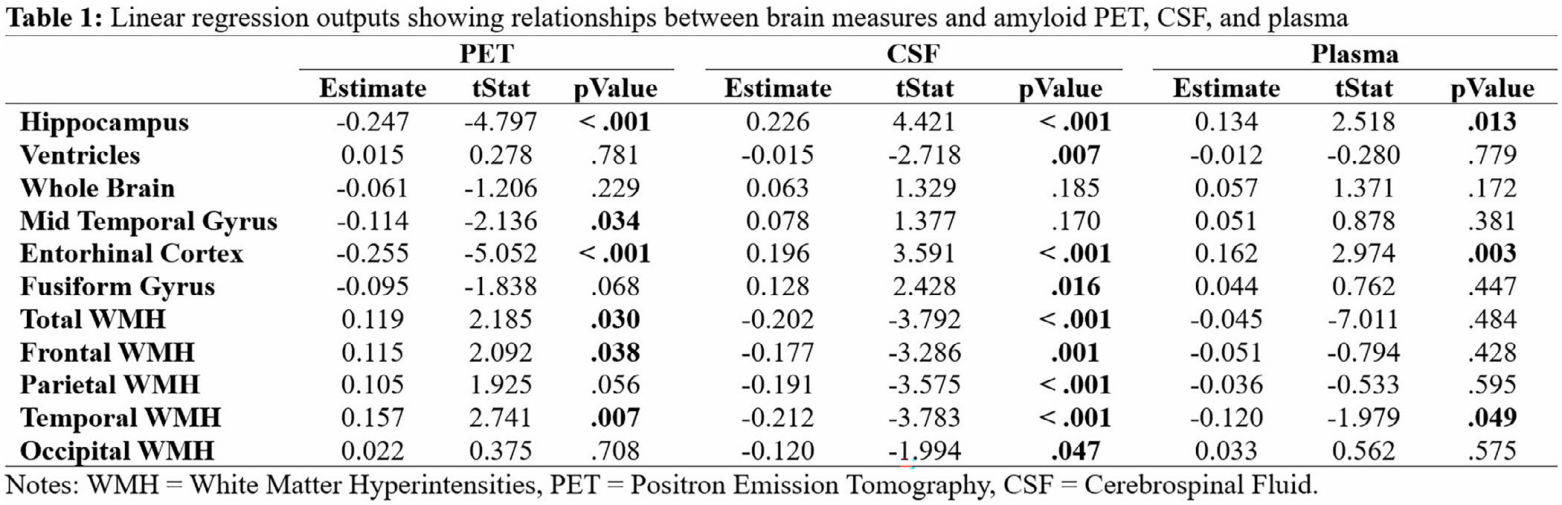# Assessing Amyloid Biomarkers in Alzheimer's Disease: Comparing the Sensitivity of PET, CSF, and Plasma to Alzheimer‐Related Brain and Cognitive Changes

**DOI:** 10.1002/alz70856_105771

**Published:** 2026-01-09

**Authors:** Jason Scully, Callista Smith, Mahsa Dadar, Cassandra Morrison

**Affiliations:** ^1^ Carleton University, Ottawa, ON, Canada; ^2^ Douglas Mental Health University Institute, Montréal, QC, Canada; ^3^ Department of Psychiatry, McGill University, Montréal, QC, Canada

## Abstract

**Background:**

With the recent availability of the new amyloid‐beta directed treatments for Alzheimer's disease (AD), accurate assessment of amyloid burden has gained increasing importance. However, limited data exists examining the sensitivity of PET, CSF, and plasma amyloid biomarkers to AD‐related brain and cognitive changes.

**Methods:**

248 participants who had MRI as well as amyloid measurements based on CSF, PET, and plasma were included from the ADNI database. Correlation analyses and linear regression models were performed to assess the intercorrelations of the three biomarker levels (i.e., CSF, PET, plasma) as well as their association with cognitive performance and brain measurements, with age, sex, and education added as covariates.

**Results:**

CSF and PET had the strongest correlation (*r* = ‐0.565, *p* < .001), followed by PET and plasma (*r* = ‐0.390, *p* < .001), and CSF and plasma (*r* = 0.338, *p* < .001, Figure 1a.). Amyloid progressively increased from CN, to MCI, to dementia (*p* < .001) when using both PET and CSF. Using plasma, however, CN participants were not significantly different from MCI participants (*p* = .10), nor did MCI participants differ from dementia participants, *p* = .36 (Figure 1b). Structural brain changes differed in their association with biomarker measurements (Table 1). For example, hippocampal volume was significantly associated with CSF amyloid (*p* < .001), PET amyloid (*p* < .001), and plasma amyloid (*p* = .01), while ventricular volume was significantly associated with CSF amyloid, (*p* = .01), but not PET (*p* = .78) or plasma (*p* = .78, Figure 2a). Global cognitive functioning (measured by the MoCA) and functional status (measured by the functional activities questionnaire) were significantly associated with CSF and PET (*p* < .001), but not plasma (*p* > .05, Figure 2b).

**Conclusion:**

Research in AD and dementia often uses CSF, PET, or plasma to discuss findings related to amyloid, which may lead to contradictory findings. These findings highlight how different measurement tools for quantifying amyloid are associated with brain and cognitive outcomes. Our findings suggest that current plasma measures of amyloid might not be sufficiently sensitive to AD‐related pathology, brain, and cognitive changes to replace PET and CSF measures.